# Global research trends on the links between the gut microbiome and cancer: a visualization analysis

**DOI:** 10.1186/s12967-022-03293-y

**Published:** 2022-02-11

**Authors:** Sa’ed H. Zyoud, Samah W. Al-Jabi, Riad Amer, Muna Shakhshir, Moyad Shahwan, Ammar A. Jairoun, Maha Akkawi, Adham Abu Taha

**Affiliations:** 1 Department of Clinical and Community Pharmacy, College of Medicine and Health Sciences, An-Najah National University, 44839 Nablus, Palestine; 2 Clinical Research Center, An-Najah National University Hospital, 44839 Nablus, Palestine; 3 Department of Hematology and Oncology, An-Najah National University Hospital, 44839 Nablus, Palestine; 4 Department of Medicine, College of Medicine and Health Sciences, An-Najah National University, 44839 Nablus, Palestine; 5 Department of Nutrition, An-Najah National University Hospital, 44839 Nablus, Palestine; 6 College of Pharmacy and Health Sciences, Ajman University, Ajman, United Arab Emirates; 7 Department of Health and Safety, Dubai Municipality, Dubai, United Arab Emirates; 8 Department of Pathology, An-Najah National University Hospital, 44839 Nablus, Palestine; 9 Department of Biomedical Sciences, College of Medicine and Health Sciences, An-Najah National University, 44839 Nablus, Palestine

**Keywords:** Microbiota, Cancer, Scopus, VOSviewer, Visualization

## Abstract

**Background:**

Significant links between the microbiota and human health have emerged in the last 20 years. A correlation has recently been demonstrated between changes in the gut microbiota and the development of cancer. This study aimed to use bibliometric analysis of the published gut microbiome and cancer literature to present the research status and summarize the hotspots for frontier studies.

**Methods:**

A literature search for research on the gut microbiome and cancer research from 2001 to 2020 was conducted using the Scopus database on 20 March 2021. VOSviewer software (version 1.6.16) was used to perform the visualization analysis.

**Results:**

From 2001 to 2020, a total of 2061 publications were retrieved. Annual publication output grew from 10 in 2001 to 486 in 2020. The USA had the largest number of publications, making the largest contribution to the field (n  = 566, 27.46%). Before 2016, most studies focused on the ‘effect of probiotics on cancer’. The latest trends showed that ‘microbiota composition and gene expression’ and ‘host-microbiome interaction in cancer immunotherapy’ would be more concerned more widely in the future.

**Conclusions:**

Research on ‘microbiota composition and gene expression’ and ‘host-microbiome interaction in cancer immunotherapy’ will continue to be the hotspot. Therefore, this study provides the trend and characteristics of the literature on the gut microbiota and cancer literature, which provided a useful bibliometric analysis for researchers to conduct further research.

## Background

Cancer is a multifaceted disease that is the second leading cause of death in the world [1]. Numerous studies in recent years have highlighted the dual role of the gut microbiota in maintaining host health [2, 3]. Gut bacteria can generate various metabolites and bioproducts that are essential for maintaining homeostasis in both the host and the gut [2]. The gut microbiota has been implicated in cancer and has been demonstrated to alter the effectiveness of anticancer drugs [4–6]. Resistance to chemo drugs or immune checkpoint inhibitors is associated with altered gut microbiota, while supplementation with different bacterial organisms restores anticancer drug responses [6]. As a result, researchers are looking at manipulating the intestinal microbiota with antibiotics, probiotics, prebiotics, or fecal transplantation to increase the effectiveness of anticancer drug effectiveness and reduce toxicity [7–9].

The status of research and scientific output on the gut microbiota and cancer is unknown. This is hypothesized and expected to be in progress, as shown in other microbiota science, such as trends in the research field of the gut microbiota [10], intestinal microbiota in obesity [11, 12], microbiome-gut-brain axis [13], gut microbiota and depression [14], microbiota of diabetes research [15], and fecal microbiota transplantation [16]. In addition, bibliometric research has recently been published in various scientific disciplines [17–25]. Bibliometric analysis differs from systematic reviews in which systematic reviews attempt to answer a specific research question based on a small number of publications [26–28]. In contrast, bibliometric analysis aims to answer a specific research question based on a large number of publications [29]. It also varies from scoping reviews, which are designed to determine the type and scope of research evidence [30, 31]. Despite these limitations, the bibliometric analysis gives a valuable overview of a field’s national and worldwide contributions to literature. It also offers baseline data, which helps to identify research gaps that might be addressed in future studies [32].

To our knowledge, only one bibliometric research was specifically based on the microbiota and restricted to gastric cancer, with data collected from the Web of Science (WoS) database [33]. As a result, to date, no comprehensive evaluation of the current literature on gut microbiota and cancer has been performed or published. Therefore, this study aimed to provide an overview of research activity on microbiota and cancer at the global level in terms of bibliometric indices. Furthermore, this study covers the latest developments, hotspots for frontier studies, and future research advancement patterns in this area. In addition, future paths and patterns in this field are forecast based on the evaluation of research output in microbiota and cancer papers.

## Methods

### Data sources

For source publication retrieval, the Scopus database was chosen as the target database. Scopus is widely recognized as one of the best online databases for bibliometric research [34, 35].

### Search strategies

We used the Scopus online database’s “Advanced search” feature and inserted the appropriate keywords to find relevant literature on microbiota and cancer from the last 2 decades (from January 2001 to December 31, 2020). To prevent bias caused by ongoing database updates, document extraction and export should be done within 1 day (March 19, 2021). Synonyms for the gut microbiota and cancer were used as in the following search strategy.

#### Step 1

To achieve the objectives of this bibliometric analysis, terms related to the microbiota entered into the Scopus engine were chosen from the literature related to the microbiota [10, 11, 13–16, 33]. The following ‘terms’ were used in the ‘Article Title’: ‘Bifidobacterium’ OR ‘dysbiosis’ OR ‘Escherichia coli’ OR ‘flora’ OR ‘Lactobacillus’ Microbiome’ OR ‘microbiota’ OR ‘microflora’ OR ‘probiotic’ OR ‘Saccharomyces’.

#### Step 2

After that, we limited the publications found in Step 1 to those that included the terms “cancer and associated terms” in their title. Terms related to cancer that were entered into the Scopus engine were extracted from the Medical Subject Headings (MeSH) from PubMed. The following ‘terms’ were entered as ‘Article Title’: ‘Neoplasia’ OR ‘Neoplasia’ OR ‘Neoplasm’ OR ‘Tumor’ OR ‘Cancer’ OR “Malignancy” OR ‘carcinoma’ OR ‘Malignancies’ OR ‘Malignant’.

### Bibliometric analysis

An Excel spreadsheet was used to collect the following data as bibliometric indicators: total number of publications, year of publication, publication types, to ten funding agencies, top ten countries, top ten institutions, top ten journals, most cancer types related to microbiota based in the top keyword, and top ten citations.

### Visualise analysis

The VOSviewer software tool (version 1.6.16) was used to build, visualize and explore networks of countries, authors, and terms, with links between items based on their cooccurrences [36, 37], was applied to create network visualization maps of the most cooccurrence terms to determine the hotspots for frontier studies, as well as the most coauthorships of countries.

## Results

### General description of the retrieved publications

In total, 2061 publications were included in this study. Articles made up 1346 of the total number of publications, accounting for 65.31% of the total number of records, making them the most popular form of literature. There were 511 reviews, representing 24.79 percent of the total. The other eight types of publications were 204 documents, representing 9.90% of the total, including letters, book chapter, notes, book, editorials, minutes of meetings, erratum, and short surveys.

### Growth and productivity trends

**Fig. 1:**
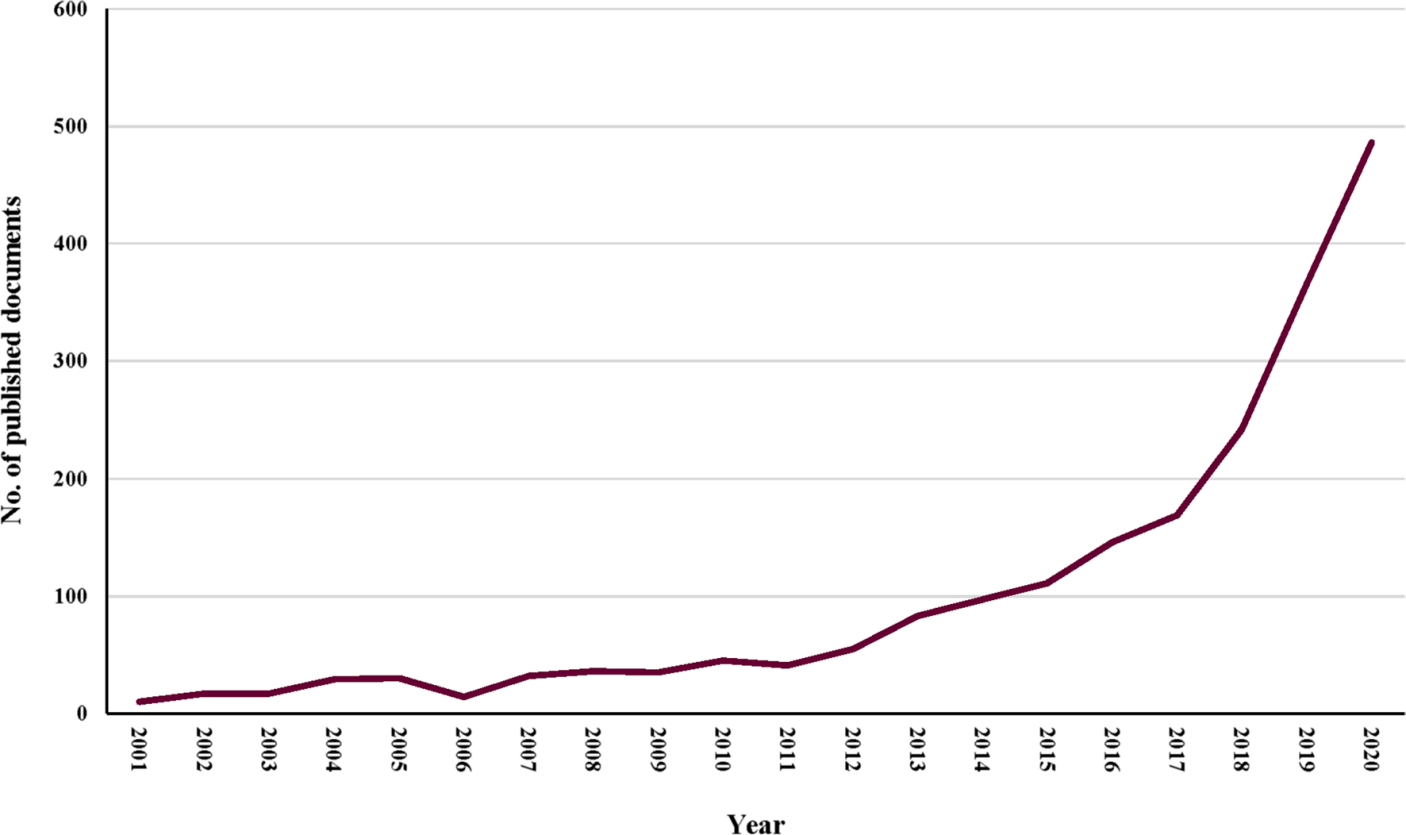
Growth trends of publications on the microbiota and cancer from 2001 to 2020

During the past 2 decades, the number of microbiota and cancer publications increased yearly, from 10 in 2001 to 486 in 2020, as shown in Fig. 1. The growth of the publication showed two stages: the first (2001–2016), which had a very slow rate of publication production, and the second (2017–2020), which had a much faster rate of publication progress.

### Top active countries

**Fig. 2:**
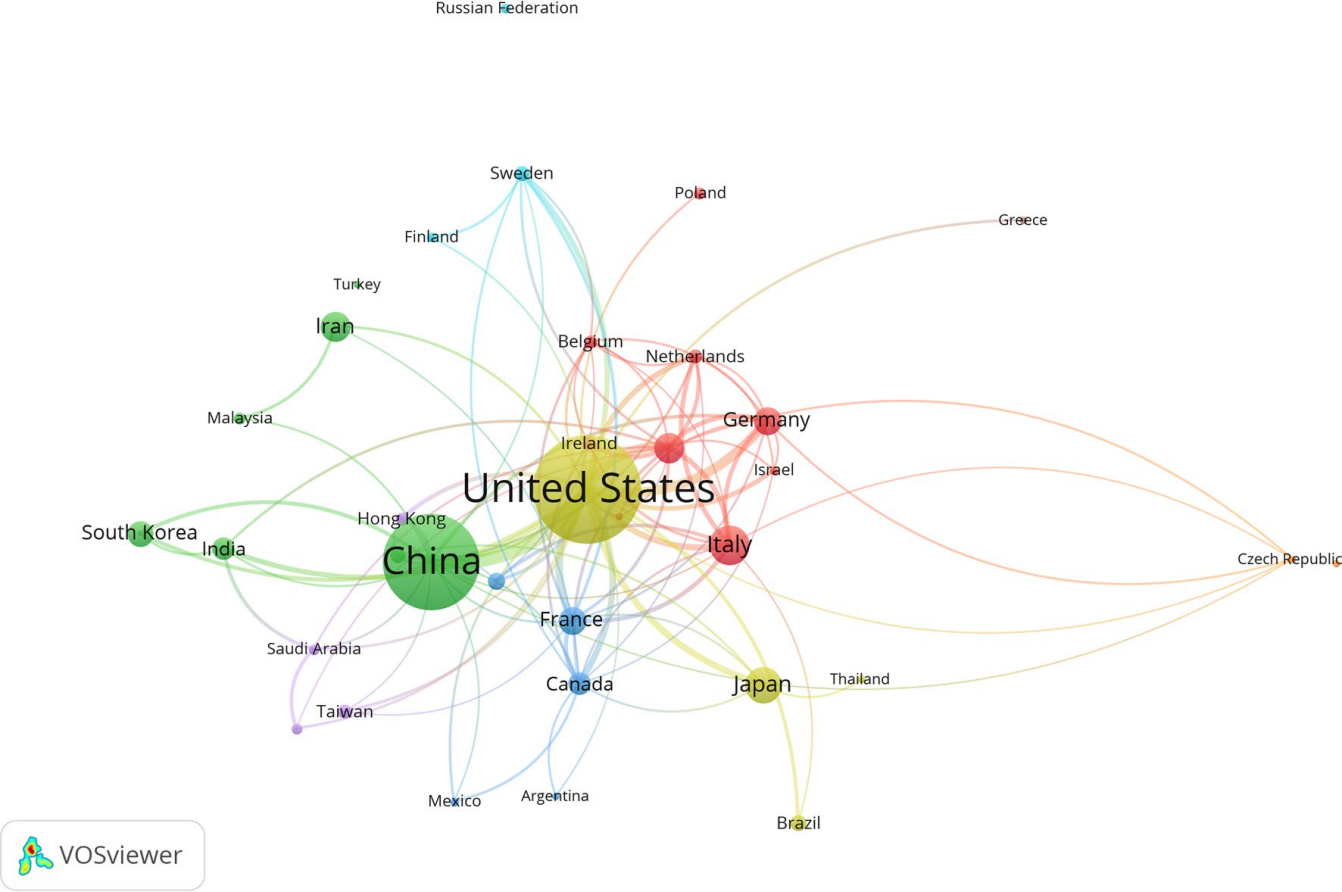
Network visualization map of international research collaboration among the leading active countries in microbiota and cancer from 2001 to 2020. This visualized map of collaborations was developed when at least 10 publications were placed for each country. There are 35 countries that reach this threshold out of 112 countries active in this field. The size of the node indicates how many publications for that country

At least 112 different countries participated in the publication of studies on the microbiota and cancer in the last 2 decades. A total of 1568 articles written in the top ten countries represented 70.8% of all studies in the related area (Table 1). The United States (n = 566) was the largest contributor, followed by China (n = 478), Italy (n = 135), Japan (n = 122) and the United Kingdom (n = 94). In addition, the United States and China had the largest number of publications involving international scholars. Figure 2 shows a network mapping chart of international research collaborations on microbiota and cancer from 2001 to 2020 among the leading participating countries. The total number of authors published on microbiota and cancer was 9102, of which 25 published more than 15 documents.

**Table 1:** List of the top 10 countries publishing research on microbiota and cancer from 2001 to 2020

Ranking	Country	Number of documents	%
1st	United States	566	27.46
2nd	China	478	23.19
3rd	Italy	135	6.55
4th	Japan	122	5.92
5th	United Kingdom	94	4.56
6th	Iran	91	4.42
7th	France	83	4.03
8th	Germany	82	3.98
9th	South Korea	74	3.59
10th	Canada	63	3.06

### Top active institutions

The Institut National de la Santé et de la Recherche Médicale (INSERM) in France had the highest number of publications among institutions worldwide, with 40 publications, which accounted for 1.94% of all publications. The National Cancer Institute of the USA was the second prolific institute with 35 (1.70%) publications, followed by the MD Anderson Cancer Center of the University of Texas in the USA with 30 (1.46%) publications, the Ministry of Education of China with 28 (1.36%) and the University of North Carolina at Chapel Hill in the USA with 28 (1.36%) publications (Table 2).

**Table 2:** List of the top 10 institutions publishing research on microbiota and cancer from 2001 to 2020

Ranking	Institute	Country	*n*	%
1st	INSERM	France	40	1.94
2nd	National Cancer Institute NCI	USA	35	1.70
3rd	University of Texas MD Anderson Cancer Center	USA	30	1.46
4th	Ministry of Education China	China	28	1.36
4th	The University of North Carolina at Chapel Hill	USA	28	1.36
6th	National Institutes of Health NIH	USA	27	1.31
6th	Tehran University of Medical Sciences	Iran	27	1.31
8th	Harvard Medical School	USA	26	1.26
9th	Tabriz University of Medical Sciences	Iran	25	1.21
10th	Zhejiang University	China	24	1.16
10th	Universite Paris-Saclay	France	24	1.16

### Top funding agencies

Of the documents that were retrieved, USA funding agencies were the most active in this field, the National Natural Science Foundation of China (n = 211; 10.24%) being the most active, followed by the National Cancer Institute (n = 186; 9.02%), National Institutes of Health (n = 163; 7.91%) and the National Institute of Diabetes and Digestive and Kidney Diseases (n = 80; 3.88%) (Table 3).

**Table 3:** The top ten funding agencies having the most publications on microbiota and cancer from 2001 to 2020

Ranking	Funding agencies	Country	Number of publication	%
1st	National Natural Science Foundation of China	China	211	10.24
2nd	National Cancer Institute	USA	186	9.02
3rd	National Institutes of Health	USA	163	7.91
4th	National Institute of Diabetes and Digestive and Kidney Diseases	USA	80	3.88
5th	Japan Society for the Promotion of Science	Japan	48	2.33
6th	National Institute of Allergy and Infectious Diseases	USA	33	1.60
7th	European Commission	European Union	30	1.46
8th	National Institute of General Medical Sciences	China	25	1.21
9th	Ministry of Education, Culture, Sports, Science and Technology	Japan	24	1.16
10th	National Research Foundation of Korea	Korea	24	1.16

### Top active journals

The top 10 journals with the most publications related to microbiota and cancer are listed in Table 4. *Scientific Reports* had the highest number of publications among journals worldwide, with 40 publications representing 1.94% of all publications. *The International Journal of Molecular Sciences* and *PloS ONE* were the second with 33 (1.6%) publications for each journal, followed by *Frontiers in Microbiology* with 27(1.31%) publications.

**Table 4:** List of the top 10 journals publishing research on microbiota and cancer from 2001 to 2020

Ranking	Journal	Frequency	%	IF^a^
1st	*Scientific Reports*	40	1.94	3.998
2nd	*International Journal of Molecular Sciences*	33	1.60	4.556
3rd	*PloS ONE*	33	1.60	2.740
4th	*Frontiers in Microbiology*	27	1.31	4.235
5th	*Cancers*	23	1.12	6.126
6th	*Frontiers in Oncology*	20	0.97	4.848
7th	*Journal of Functional Foods*	19	0.92	3.701
8th	*Gut*	18	0.87	19.819
9th	*Gut Microbes*	18	0.87	7.740
10th	*Nutrition and Cancer*	17	0.82	2.363

*SCR* standard competition ranking; *IF* impact factor

^a^Impact factors based on the 2019 Journal Citation Reports 2019 from Clarivate Analytics

### Top-cited publications

Table 5 summarizes the top ten most cited papers in the microbiota and cancer during the last 2 decades based on total citations. The top 10 highest citations ranged from 1378 to 441 [38–47]. Routy et al. [44], published in *Science* in 2018, had the highest overall citation frequency (number of citations = 1378) among the top ten publications with total citation frequency.

**Table 5:** Top cited list of the top 10 high cited papers related to microbiota and cancer from 2001 to 2020

Ranking	Authors	Year	Source title	Cited by
1st	Routy et al. [44]	2018	*Science*	1378
2nd	Arthur et al. [38]	2012	*Science*	1023
3rd	Louis et al. [42]	2014	*Nature Reviews Microbiology*	966
4th	Schwabe and Jobin [45]	2013	*Nature Reviews Cancer*	702
5th	Dapito et al. [40]	2012	*Cancer Cell*	592
6th	Wang et al. [47]	2012	*ISME Journal*	555
7th	Sobhani [46]	2011	*PLoS ONE*	493
8th	Martin et al. [43]	2004	*Gastroenterology*	481
9th	Chen et al. [39]	2012	*PLoS ONE*	449
10th	Garret [41]	2015	*Science*	441

### Hotspots for frontier studies

**Fig. 3:**
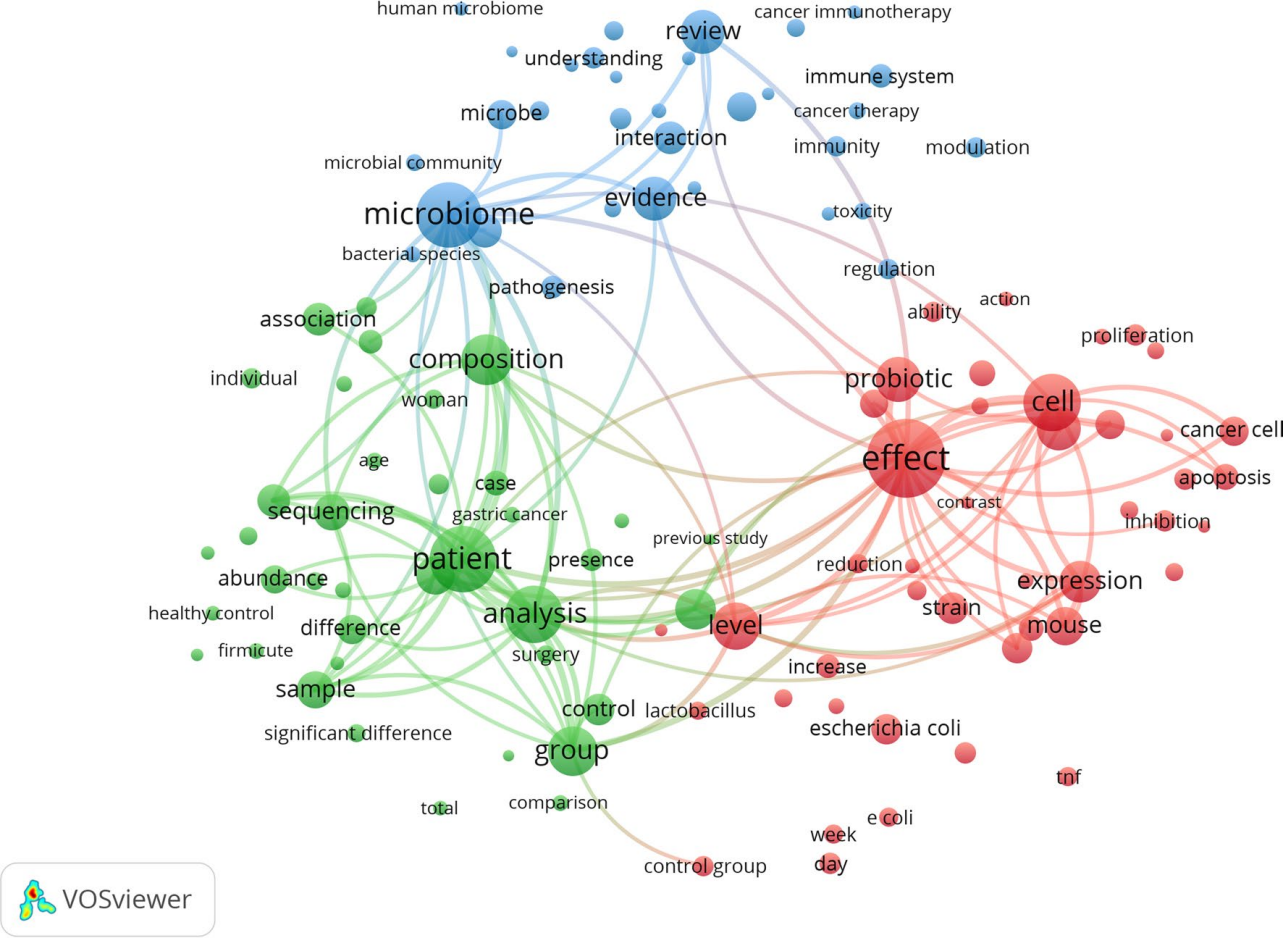
Network visualization map of terms in title/abstract fields of publications related to microbiota and cancer from 2001 to 2020. This visualized map of terms was developed when the minimum-term occurrences were placed at least 50 times. There are 109 terms that reach this threshold out of 34,784 in this field, which were divided into three clusters and colored differently. The size of the node indicates how many publications use that term

**Fig. 4:**
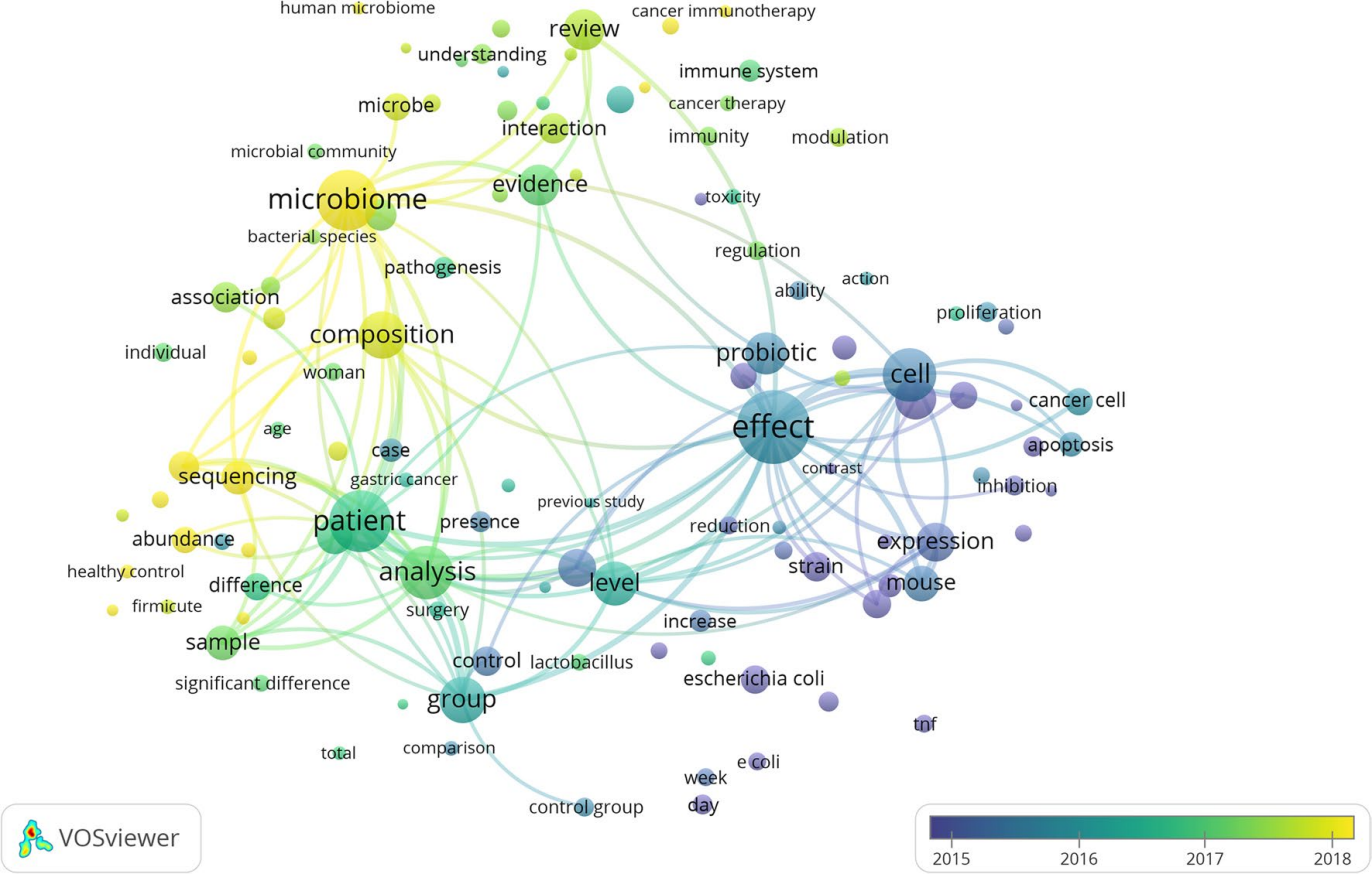
Network visualization map of terms in the title/abstract and their distribution according to the mean frequency of appearance. The blue terms emerged first, followed by the yellow and green terms that appeared later

Terms with a minimum number of occurrences greater than 50 in all included publications were analyzed using VOSviewer. There are 109 terms that reach this threshold out of 34,784 in this field, which were divided into three clusters and colored differently. The three clusters are “microbiota composition and gene expression” (cluster 1, green), “effect of probiotics on cancer” (cluster 2, red), and “host-microbiome interaction in cancer immunotherapy” (cluster 3, blue); (Fig. 3). In cluster 1, the most striking keywords are sequence, abundance, composition, healthy control, and patient. In cluster 2, the most common keywords are effect, probiotic, ability, apoptosis, proliferation, reduction, cancer cell, and inhibition. In cluster 3, the keywords with the most repetition are microbiome, evidence, pathogenesis, cancer, immunotherapy, interaction, cancer therapy, and immunity. These results demonstrated that the most prominent fields of microbiota and cancer included 3 directions during the past 20 years. Keywords were color coded by VOSviewer based on the average time they appeared in the 2061 related publications (Fig. 4). The terms in blue appeared earlier, and those in yellow and green appeared later. Before 2016, most studies focused on the ‘effect of probiotics on cancer’. The latest trends showed that ‘microbiota composition and gene expression’ and ‘host-microbiome interaction in cancer immunotherapy’ would be more concerned more widely in the future.

### The most frequent cancer types encountered in the retrieved literature

Table 6 lists the most frequent keywords related to cancer types occurrences in microbiota literature from 2001 to 2020. The top keywords indicate that colorectal cancer is strongly related to microbiota in the literature, followed by breast cancer, stomach cancer, and lung cancer (Table 6).

**Table 6:** List of most frequent keywords related to cancer types occurrences in microbiota literature from 2001 to 2020

Cancer types	Frequency	%
Colorectal cancer	559	27.12
Colorectal neoplasms	347	16.84
Colorectal tumor	280	13.59
Colon cancer	191	9.27
Colonic neoplasms	150	7.28
Breast cancer	124	6.02
Colon tumor	111	5.39
Stomach cancer	103	5.00
Breast neoplasms	79	3.83
Gastrointestinal cancers	78	3.78
Lung cancer	78	3.78

## Discussion

This is the first bibliometric study to evaluate and visualize research of the gut microbiota in the field of oncology. A total of 2061 publications originating from the Scopus database were analyzed. We offer a detailed analysis of global trends and hotspots in gut microbiota and cancer research over the past 2 decades. According to our study, publications have increased rapidly since 2017.

Previous research on microbiota and gastric cancer [33] found findings that differed from those presented in our study (196 documents worldwide from 2000 to 2019). The inconsistency was attributable to (1) the various databases used to access the publications and (2) the different research domains analyzed. Zhang et al. [33] study was carried out using WoS and was limited to gastric cancer. In our study, the Scopus database used more comprehensive terms related to gut microbiota and cancer without limiting the results to any particular cancer. We conducted our bibliometric research using the Scopus database, which Elsevier owns. According to several studies, Scopus is the world’s largest database of abstracts and citations from peer-reviewed scientific literature [48–50]. Various scholars for bibliometric evaluation of various fields of science [48–52]. Scopus is a multidisciplinary database that has more indexed journals than PubMed and WoS [53–55].

The United States was the leading contributor in this field with 566 publications, followed by China, Italy, Japan, and the United Kingdom. Research success in these countries can be linked to a diverse group of researchers with expertise in this area and a significant amount of financial funding for researchers. This research productivity is not unexpected, considering that it is in countries with stronger infrastructures, more abundant scientific services, and a long history in the general study of the microbiome. Many experts in the fields of microbiology, oncology, and gastroenterology (eg., Marchesi JR, Jobin C, Trinchieri G, Perdigón G, Qin H, Gao R, Wargo JA, White JR, Zitvogel L, Ahn J, Fujimori M, Goedert JJ, Mahdavi M, Morrow CD, Oue N, Sentani K, Sobhani I, Yasui W, Yu J, Zitvogel L, and others) are gaining interest in the pathological function and host-microbiome relationship in cancer as an evolving forum to evaluate the diagnosis and effective therapeutic intervention of these ailments, which is possibly contributing to the increase in research productivity [56–68].

Furthermore, the increase in the number of publications on microbiota and cancer may be linked to many hot topics published during this time frame, exposing innovative theories that lead to new fields of research. These findings suggest new therapeutic and diagnostic concepts for various disorders in the field of oncology [38–47, 69–73] such as “effect of probiotics on cancer”, “microbiota composition and gene expression” and “host-microbiome interaction in cancer immunotherapy”.

The results of our study indicate that the most highly cited microbiota and cancer publications emphasized on a variety of subtopics close to the study hotspots in co-occurring terms, which is a strong trend emerging from the results. The article most cited was Routy et al. [44] entitled ‘Gut microbiome influences the efficacy of PD-1-based immunotherapy against epithelial tumors’, published in *Science* in 2018, with 1,378 total citations. This study reported that the maintenance of healthy gut flora could affect patient responses to cancer immunotherapy and help patients combat cancer. The second paper was by Arthur et al. [38] from *Science*. The complex effects of inflammation on the microbial composition/activity and the host’s ability to defend itself from a dysbiotic microbiota were illustrated in this report [38]. The third most cited paper is a study by Louis et al. [42] in *Nature Reviews Microbiology* as a review, which discusses the relationship between microbial metabolism, diet, and colorectal cancer, and argues that the cumulative effects of microbial metabolites should be considered to help predict and prevent cancer progression.

### Strengths and limitations

The current study is the first bibliometric study of its kind to look at this emerging topic and provide comprehensive information on research trends and hotspots in this field. Three elements of current research are unique: (1) the use of Scopus, the largest database available; (2) the use of terms related to the gut microbiota and cancer comprehensively; and (3) it is global in scope. Finally, the findings of our research have some limitations. First, this study’s analysis is based on papers found in the Scopus database. Although this database contains most of the majority of research papers on gut microbiota and cancer, other databases such as PubMed and WoS may have some publications related to our topic, which is a weakness of this article. Second, the fact that we only included publications on cancer or gut microbiota in the article title has an inherent flaw; our experience has shown that including search items in the abstract has a much lower sensitivity and would have only found a small number of additional papers, if any. If we include terms from the abstract in our search query without any restrictions, we will get a lot of irrelevant publications that are not related to our subject. Despite these limitations, we believe that the findings provide a reliable representation of the performance of the gut microbiota and cancer research at a global level.

## Conclusions

This study shows the current status of microbiota and cancer on a global level and the hottest directions. According to the pattern in recent years, there will be a rise in the number of publications in this field. Until now, the United States and China have made the most significant contributions in the field of microbiota and cancer. Research on ‘microbiota composition and gene expression’ and ‘host-microbiome interaction in cancer immunotherapy’ will continue to be the hotspot. This serves as a starting point for further debate, while also emphasizing the need for further analysis.

## Data Availability

The data sets generated and/or analysed during the current study are available upon request from the corresponding authors.

## Abbreviations

IFs: Impact factors
JCR: Journal citation reports
MeSH: Medical subject headings

## References

[1] GBD 2015 Mortality and Causes of Death Collaborators (2016). Global, regional, and national life expectancy, all-cause mortality, and cause-specific mortality for 249 causes of death, 1980–2015: a systematic analysis for the Global Burden of Disease Study 2015. Lancet.

[2] Vivarelli S, Salemi R, Candido S, Falzone L, Santagati M, Stefani S, Torino F, Banna GL, Tonini G, Libra M (2019). Gut microbiota and cancer: from pathogenesis to therapy. Cancers.

[3] Gaucher L, Adda L, Séjourné A, Joachim C, Guillaume C, Poulet C, Liabeuf S, Gras-Champel V, Masmoudi K, Houessinon A (2021). Associations between dysbiosis-inducing drugs, overall survival and tumor response in patients treated with immune checkpoint inhibitors. Ther Adv Med Oncol.

[4] Iida N, Dzutsev A, Stewart CA, Smith L, Bouladoux N, Weingarten RA, Molina DA, Salcedo R, Back T, Cramer S (2013). Commensal bacteria control cancer response to therapy by modulating the tumor microenvironment. Science.

[5] Butterfield LH, Kaufman HL, Marincola FM (2021). Cancer immunotherapy principles and practice.

[6] Cheng WY, Wu CY, Yu J (2020). The role of gut microbiota in cancer treatment: friend or foe?. Gut.

[7] Gori S, Inno A, Belluomini L, Bocus P, Bisoffi Z, Russo A, Arcaro G (2019). Gut microbiota and cancer: how gut microbiota modulates activity, efficacy and toxicity of antitumoral therapy. Crit Rev Oncol Hematol.

[8] Raza MH, Gul K, Arshad A, Riaz N, Waheed U, Rauf A, Aldakheel F, Alduraywish S, Rehman MU, Abdullah M (2019). Microbiota in cancer development and treatment. J Cancer Res Clin Oncol.

[9] Borella F, Carosso AR, Cosma S, Preti M, Collemi G, Cassoni P, Bertero L, Benedetto C (2021). Gut microbiota and gynecological cancers: a summary of pathogenetic mechanisms and future directions. ACS Infect Dis.

[10] Yue YY, Fan XY, Zhang Q, Lu YP, Wu S, Wang S, Yu M, Cui CW, Sun ZR (2020). Bibliometric analysis of subject trends and knowledge structures of gut microbiota. World J Clin Cases.

[11] Yao H, Wan JY, Wang CZ, Li L, Wang J, Li Y, Huang WH, Zeng J, Wang Q, Yuan CS (2018). Bibliometric analysis of research on the role of intestinal microbiota in obesity. PeerJ.

[12] Ejtahed HS, Tabatabaei-Malazy O, Soroush AR, Hasani-Ranjbar S, Siadat SD, Raes J, Larijani B (2019). Worldwide trends in scientific publications on association of gut microbiota with obesity. Iran J Basic Med Sci.

[13] Zyoud SH, Smale S, Waring WS, Sweileh WM, Al-Jabi SW (2019). Global research trends in microbiome-gut-brain axis during 2009–2018: a bibliometric and visualized study. BMC Gastroenterol.

[14] Zhu X, Hu J, Deng S, Tan Y, Qiu C, Zhang M, Ni X, Lu H, Wang Z, Li L (2020). Bibliometric and visual analysis of research on the links between the gut microbiota and depression from 1999 to 2019. Front Psychiatry.

[15] Tian J, Li M, Lian F, Tong X (2017). The hundred most-cited publications in microbiota of diabetes research: a bibliometric analysis. Medicine.

[16] Li Y, Zou Z, Bian X, Huang Y, Wang Y, Yang C, Zhao J, Xie L (2019). Fecal microbiota transplantation research output from 2004 to 2017: a bibliometric analysis. PeerJ.

[17] Bougioukas KI, Vounzoulaki E, Mantsiou CD, Papanastasiou GD, Savvides ED, Ntzani EE, Haidich AB (2021). Global mapping of overviews of systematic reviews in healthcare published between 2000 and 2020: a bibliometric analysis. J Clin Epidemiol.

[18] Du YQ, Zhu GD, Cao J, Huang JY (2021). Research supporting malaria control and elimination in China over four decades: a bibliometric analysis of academic articles published in chinese from 1980 to 2019. Malar J.

[19] Gonzalez-Alcaide G, Palacios-Fernandez S, Ramos-Rincon JM (2021). Thematic research clusters in very old populations (>/= 80 years): a bibliometric approach. BMC Geriatr.

[20] Guleid FH, Oyando R, Kabia E, Mumbi A, Akech S, Barasa E (2021). A bibliometric analysis of COVID-19 research in Africa. BMJ Glob Health.

[21] Sahin S, Sivri N, Akpinar I, Cincin ZB, Sonmez VZ (2021). A comprehensive bibliometric overview: antibiotic resistance and *Escherichia**coli* in natural water. Environ Sci Pollut Res Int.

[22] Sweileh WM (2021). Contribution of researchers in the Arab region to peer-reviewed literature on mental health and well-being of university students. Int J Ment Health Syst.

[23] Torres RT, Carvalho J, Cunha MV, Serrano E, Palmeira JD, Fonseca C (2021). Temporal and geographical research trends of antimicrobial resistance in wildlife—a bibliometric analysis. One Health.

[24] Wang K, Duan W, Duan Y, Yu Y, Chen X, Xu Y, Chen H, Huang H, Xiong B (2020). A bibliometric insight of genetic factors in ASD: emerging trends and new developments. Brain Sci.

[25] Zyoud SH (2021). The Arab region’s contribution to global COVID-19 research: bibliometric and visualization analysis. Global Health.

[26] Møller AM, Myles PS (2016). What makes a good systematic review and meta-analysis?. Br J Anaesth.

[27] O’Gorman CS, Macken AP, Cullen W, Saunders J, Dunne C, Higgins MF (2013). What are the differences between a literature search, a literature review, a systematic review and a meta-analysis? And why is a systematic review considered to be so good?. Ir Med J.

[28] Peters MD, Godfrey CM, Khalil H, McInerney P, Parker D, Soares CB (2015). Guidance for conducting systematic scoping reviews. Int J Evid Based Healthc.

[29] Wallin JA (2005). Bibliometric methods: pitfalls and possibilities. Basic Clin Pharmacol Toxicol.

[30] Levac D, Colquhoun H, O'Brien KK (2010). Scoping studies: advancing the methodology. Implement Sci.

[31] Grant MJ, Booth A (2009). A typology of reviews: an analysis of 14 review types and associated methodologies. Health Info Libr J.

[32] Sweileh WM, Moh'd Mansour A (2020). Bibliometric analysis of global research output on antimicrobial resistance in the environment (2000–2019). Glob Health Res Policy.

[33] Zhang T, Yin X, Yang X, Man J, He Q, Wu Q, Lu M (2020). Research trends on the relationship between microbiota and gastric cancer: a bibliometric analysis from 2000 to 2019. J Cancer.

[34] Baas J, Schotten M, Plume A, Côté G, Karimi R (2020). Scopus as a curated, high-quality bibliometric data source for academic research in quantitative science studies. Quant Sci Stud.

[35] Sweileh WM (2020). Bibliometric analysis of scientific publications on “sustainable development goals” with emphasis on “good health and well-being” goal (2015–2019). Global Health.

[36] van Eck NJ, Waltman L (2010). Software survey: VOSviewer, a computer program for bibliometric mapping. Scientometrics.

[37] van Eck NJ, Waltman L (2017). Citation-based clustering of publications using CitNetExplorer and VOSviewer. Scientometrics.

[38] Arthur JC, Perez-Chanona E, Mühlbauer M, Tomkovich S, Uronis JM, Fan TJ, Campbell BJ, Abujamel T, Dogan B, Rogers AB (2012). Intestinal inflammation targets cancer-inducing activity of the microbiota. Science.

[39] Chen W, Liu F, Ling Z, Tong X, Xiang C (2012). Human intestinal lumen and mucosa-associated microbiota in patients with colorectal cancer. PLoS ONE.

[40] Dapito DH, Mencin A, Gwak GY, Pradere JP, Jang MK, Mederacke I, Caviglia JM, Khiabanian H, Adeyemi A, Bataller R (2012). Promotion of hepatocellular carcinoma by the intestinal microbiota and TLR4. Cancer Cell.

[41] Garrett WS (2015). Cancer and the microbiota. Science.

[42] Louis P, Hold GL, Flint HJ (2014). The gut microbiota, bacterial metabolites and colorectal cancer. Nat Rev Microbiol.

[43] Martin HM, Campbell BJ, Hart CA, Mpofu C, Nayar M, Singh R, Englyst H, Williams HF, Rhodes JM (2004). Enhanced Escherichia coli adherence and invasion in Crohn’s disease and colon cancer. Gastroenterology.

[44] Routy B, Le Chatelier E, Derosa L, Duong CPM, Alou MT, Daillère R, Fluckiger A, Messaoudene M, Rauber C, Roberti MP (2018). Gut microbiome influences efficacy of PD-1-based immunotherapy against epithelial tumors. Science.

[45] Schwabe RF, Jobin C (2013). The microbiome and cancer. Nat Rev Cancer.

[46] Sobhani I, Tap J, Roudot-Thoraval F, Roperch JP, Letulle S, Langella P, Corthier G, Van Nhieu JT, Furet JP (2011). Microbial dysbiosis in colorectal cancer (CRC) patients. PLoS ONE.

[47] Wang T, Cai G, Qiu Y, Fei N, Zhang M, Pang X, Jia W, Cai S, Zhao L (2012). Structural segregation of gut microbiota between colorectal cancer patients and healthy volunteers. ISME J.

[48] AlRyalat SAS, Malkawi LW, Momani SM (2019). Comparing bibliometric analysis using PubMed, Scopus, and Web of science databases. J Vis Exp.

[49] Zyoud SH, Al-Jabi SW (2020). Mapping the situation of research on coronavirus disease-19 (COVID-19): a preliminary bibliometric analysis during the early stage of the outbreak. BMC Infect Dis.

[50] Sweileh WM (2020). Bibliometric analysis of global scientific literature on vaccine hesitancy in peer-reviewed journals (1990–2019). BMC Public Health.

[51] Sweileh WM (2021). Bibliometric analysis of peer-reviewed literature on antimicrobial stewardship from 1990 to 2019. Global Health.

[52] Briones-Bitar J, Carrión-Mero P, Montalván-Burbano N, Morante-Carballo F (2020). Rockfall research: a bibliometric analysis and future trends. Geosciences.

[53] Falagas ME, Pitsouni EI, Malietzis GA, Pappas G (2008). Comparison of PubMed, Scopus, Web of Science, and Google Scholar: strengths and weaknesses. FASEB J.

[54] Kulkarni AV, Aziz B, Shams I, Busse JW (2009). Comparisons of citations in Web of Science, Scopus, and Google Scholar for articles published in general medical journals. JAMA.

[55] Mongeon P, Paul-Hus A (2015). The journal coverage of Web of Science and Scopus: a comparative analysis. Scientometrics.

[56] Ahn J, Sinha R, Pei Z, Dominianni C, Wu J, Shi J, Goedert JJ, Hayes RB, Yang L (2013). Human gut microbiome and risk for colorectal cancer. J Natl Cancer Inst.

[57] Berkovic MC, Cigrovski V, Bilic-Curcic I, Mrzljak A (2020). What is the gut feeling telling us about physical activity in colorectal carcinogenesis?. World J Clin Cases.

[58] Contreras AV, Cocom-Chan B, Hernandez-Montes G, Portillo-Bobadilla T, Resendis-Antonio O (2016). Host-microbiome interaction and cancer: potential application in precision medicine. Front Physiol.

[59] Gupta A, Saha S, Khanna S (2020). Therapies to modulate gut microbiota: past, present and future. World J Gastroenterol.

[60] Huang QY, Yao F, Zhou CR, Huang XY, Wang Q, Long H, Wu QM (2020). Role of gut microbiome in regulating the effectiveness of metformin in reducing colorectal cancer in type 2 diabetes. World J Clin Cases.

[61] Malla MA, Dubey A, Kumar A, Yadav S, Hashem A, Abd Allah EF (2018). Exploring the human microbiome: the potential future role of next-generation sequencing in disease diagnosis and treatment. Front Immunol.

[62] Roy S, Trinchieri G (2017). Microbiota: a key orchestrator of cancer therapy. Nat Rev Cancer.

[63] Sabit H, Cevik E, Tombuloglu H (2019). Colorectal cancer: the epigenetic role of microbiome. World J Clin Cases.

[64] Sears CL, Garrett WS (2014). Microbes, microbiota, and colon cancer. Cell Host Microbe.

[65] Tlaskalová-Hogenová H, Stěpánková R, Kozáková H, Hudcovic T, Vannucci L, Tučková L, Rossmann P, Hrnčíř T, Kverka M, Zákostelská Z (2011). The role of gut microbiota (commensal bacteria) and the mucosal barrier in the pathogenesis of inflammatory and autoimmune diseases and cancer: contribution of germ-free and gnotobiotic animal models of human diseases. Cell Mol Immunol.

[66] Wei AL, Li M, Li GQ, Wang X, Hu WM, Li ZL, Yuan J, Liu HY, Zhou LL, Li K (2020). Oral microbiome and pancreatic cancer. World J Gastroenterol.

[67] Wollowski I, Rechkemmer G, Pool-Zobel BL (2001). Protective role of probiotics and prebiotics in colon cancer. Am J Clin Nutr.

[68] Zeller G, Tap J, Voigt AY, Sunagawa S, Kultima JR, Costea PI, Amiot A, Böhm J, Brunetti F, Habermann N (2014). Potential of fecal microbiota for early-stage detection of colorectal cancer. Mol Syst Biol.

[69] Elkrief A, Derosa L, Zitvogel L, Kroemer G, Routy B (2019). The intimate relationship between gut microbiota and cancer immunotherapy. Gut Microbes.

[70] Kujawska M, Jodynis-Liebert J (2020). Potential of the ellagic acid-derived gut microbiota metabolite—Urolithin A in gastrointestinal protection. World J Gastroenterol.

[71] Russo E, Nannini G, Dinu M, Pagliai G, Sofi F, Amedei A (2020). Exploring the food-gut axis in immunotherapy response of cancer patients. World J Gastroenterol.

[72] Yan C, Tu XX, Wu W, Tong Z, Liu LL, Zheng Y, Jiang WQ, Zhao P, Fang WJ, Zhang HY (2019). Antibiotics and immunotherapy in gastrointestinal tumors: friend or foe?. World J Clin Cases.

[73] Yi M, Jiao D, Qin S, Chu Q, Li A, Wu K (2019). Manipulating gut microbiota composition to enhance the therapeutic effect of cancer immunotherapy. Integr Cancer Ther.

